# Supplementary material for: The Utility of Urinary Titin to Diagnose and Predict the Prognosis of Acute Myocardial Infarction
Source: Int J Mol Sci. 2024 Jan 1;25(1):573. doi: 10.3390/ijms25010573 (PMC10778763; doi:10.3390/ijms25010573)
Supplement: Supplementary file 1 [file ijms-25-00573-s001.zip › ijms-2740560-supplementary.pdf]

Supplementary Table S1. Association between urinary titin and parameters in non-AMI patients and AMI patients. In AMI patient group, urinary titin was correlated with CK-MB and hsTnI. However, non-AMI patient group has no correlation with them.

Wilcoxon rank sum test

	Non-AMI(n=32)		AMI(n=51)	
	Urinary Titin (pmol/mgCr)	p-value	Urinary Titin (pmol/mgCr)	p-value
Sex		0.044		0.347
Male	6.33 (4.07-13.79)		6.31 (3.28-13.46)	
Femele	3.68 (2.80-5.89)		9.28 (4.38-17.19)	

Spearman's rank correlation

	Non-AMI(n=32)		AMI(n=51)	
	**rs**	**p-value**	**rs**	**p-value**
Age	0.092	0.617	0.262	0.063
BMI	-0.019	0.920	0.146	0.312
Systolic blood pressure	-0.348	0.059	0.221	0.119
Diastolic blood pressure	-0.206	0.274	0.052	0.718
SpO_2	-0.302	0.099	-0.129	0.368
CK	0.510	0.003	0.599	<0.001
CK-MB	0.213	0.243	0.314	0.025
Serum hs-Troponin I	0.201	0.287	0.525	<0.001
BUN	0.177	0.333	0.381	0.006
BNP	0.277	0.154	0.599	<0.001
eGFR	-0.165	0.367	-0.217	0.127
